# Autism common variants associated with white matter alterations at birth: cross-sectional fixel-based analyses of 221 European term-born neonates from the developing human connectome project

**DOI:** 10.1038/s41398-025-03252-3

**Published:** 2025-02-04

**Authors:** Hai Le, Alexandra F. Bonthrone, Alena Uus, Daphna Fenchel, Alexandra Lautarescu, Konstantina Dimitrakopoulou, A. David Edwards, Joseph V. Hajnal, Serena J. Counsell, Lucilio Cordero-Grande, Daan Christiaens, Dafnis Batalle, Maximilian Pietsch, Anthony N. Price, Hamel Patel, Charles Curtis, Harriet Cullen, Maria Deprez, Jacques-Donald Tournier

**Affiliations:** 1 Research Department of Early Life Imaging, School of Biomedical Engineering and Imaging Sciences, King’s College London, London, UK; 2 Department of Forensic and Neurodevelopmental Sciences, Institute of Psychiatry, Psychology and Neuroscience, King’s College London, London, UK; 3 Translational Bioinformatics Platform, NIHR Biomedical Research Centre, Guy’s and St. Thomas’ NHS Foundation Trust and King’s College London, London, UK; 4 Biomedical Image Technologies, ETSI Telecomunicación, Universidad Politécnica de Madrid & CIBER-BBN, ISCIII, Madrid, Spain; 5 NIHR BioResource Centre Maudsley, NIHR Maudsley Biomedical Research Centre at South London and Maudsley NHS Foundation Trust & Institute of Psychiatry, Psychology and Neuroscience, King’s College London, London, UK; 6 Social Genetic & Developmental Psychiatry Centre, Institute of Psychiatry, Psychology and Neuroscience, King’s College London, London, UK; 7 Department of Medical and Molecular Genetics, School of Basic and Medical Biosciences, King’s College London, London, UK

**Keywords:** Clinical genetics, Neuroscience

## Abstract

Increasing lines of evidence suggest white matter (WM) structural changes associated with autism can be detected in the first year of life. Despite the condition having high heritability, the relationship between autism common genetic variants and WM changes during this period remains unclear. By employing advanced regional and whole-brain fixel-based analysis, the current study investigated the association between autism polygenic scores (PS) and WM microscopic fibre density and macrostructural morphology in 221 term-born infants of European ancestry from the developing Human Connectome Project. The results suggest greater tract mean fibre-bundle cross-section of the left superior corona radiata is associated with higher autism PS. Subsequent exploratory enrichment analysis revealed that the autism risk single nucleotide polymorphisms most associated with the imaging phenotype may have roles in neuronal cellular components. Together, these findings suggest a possible link between autism common variants and early WM development.

## Introduction

Early white matter (WM) structural alterations have been identified in children with a diagnosis of autism [1–4], but the relationship between the anatomical variation and the condition remains poorly understood. Retrospective analyses have revealed a generalised WM volume increase in autistic children at two years of age [2, 3, 5]. Similarly, diffusion tensor imaging (DTI) studies have reported microstructural differences within the first year of life in infants who are later diagnosed with autism both globally [6] and across multiple WM tracts, including the corpus callosum [4], corticospinal tract [6], cingulum, superior longitudinal, uncinate and arcuate fasciculi [7–9]. Wolff et al. showed variations in callosal and cerebellar WM may also be associated with restricted and repetitive behaviour and sensory responsiveness in toddlers with autism diagnoses [10, 11]. Collectively, the literature suggests WM development may play an important role in this condition.

WM maturation appears most dynamic throughout infancy, followed by subtler changes during adolescence and adulthood [12, 13]. This is characterised by an orchestrated sequence of biological events including axonal growth, proliferation of glial progenitors and myelination. By the third trimester of pregnancy, almost all major WM tracts are detectable using diffusion imaging. Each fibre tract appears to follow a distinct spatiotemporal maturational trajectory [14], with the corpus callosum and limbic fibres developing first and association fibres last [15]. After birth, WM tracts continue to grow asynchronously, with callosal projections and cortico-spinal tracts reaching peak maturation before frontotemporal connections [16].

WM microstructure is most widely assessed using DTI parameters. However, in regions of multiple or crossing fibres (estimated in about 90% of the adult brain [17]), the tensor model remains simplistic and inadequate [18]. By employing advanced diffusion acquisition protocols that utilise multi-directional and high b-value gradient schemes [19], one can fit more complex models to decouple the intravoxel fibre heterogeneity. Multi-shell multi-tissue constrained spherical deconvolution (MSMT-CSD) [20] and the related fixel-based analysis (FBA) [21] are well-established approaches used to estimate tissue volume fractions and examine properties of “individual ***fi***bre populations within a vo***xel***”. Here, FBA gives information about the fibre’s micro- and macrostructural organisation via local fibre density and fibre cross-section metrics, the combination of which can represent the fibre’s ability to relay information [21]. Given the heterogeneous landscape of WM development in the neonatal brain, the ability to disentangle individual fibre populations within a voxel may be useful. To our knowledge, while the FBA framework has been applied to adolescence and adult autism samples [22–24], no studies to date have examined the FBA framework in infants at elevated likelihood for autism, or who are later diagnosed with autism.

Evidence suggests autism common genetic variants and candidate genes may have important roles in foetal corticogenesis [25, 26]. At the molecular level, autism risk genes appear to regulate brain-wide expression of microglial, astrocyte, neuro-immune and synaptic genes [27]. WM microstructure variations also appear to be strongly influenced by genetics during early life [28]. Examination of DTI metrics indicates heritability estimates vary both temporally (higher heritability estimates in infants compared with that in children and adults) and regionally (higher heritability in early maturing regions than in later ones) [29]. Cumulatively, genetic variation may have a significant influence on normal WM development, particularly during infancy, when the genetic expression landscape is dynamic and under tight regulatory control. Disruption of normal development during this critical window as a result of either genetic or environmental factors may have significant effects on normal WM development and function [30].

Given the evidence of the important role of autism common genetic variants on early brain development, the current work aimed to explore the relationship between the genetic variants and WM structural alterations in a general neonatal population. Specifically, by employing the fixel-based analysis, we examined the association between autism polygenic score and WM micro- and macrostructural organisation in a cohort of term-born neonates.

## Method

The current study utilises high-quality magnetic resonance imaging (MRI) and corresponding genetic data from infants recruited as part of the developing Human Connectome Project (dHCP). The inclusion criteria for imaging were live infants between 23 and 44 weeks of gestational age (GA), estimated from the mother’s last menstrual period and confirmed where possible by ultrasound. On the other hand, infants were excluded if they were not suitable for MRI (e.g., too unwell to tolerate the scanning period) or proper communication about the trial could not be conveyed to the parents (e.g., language difficulties). The dHCP was conducted according to the principles of the Declaration of Helsinki and ethical approval was given from the UK National Research Ethics Service. Written parental consent was provided for all individuals. Both the MRI and genetic data are available publicly as part of the 3rd neonatal data release [31].

### Diffusion data acquisition

**Fig. 1:**
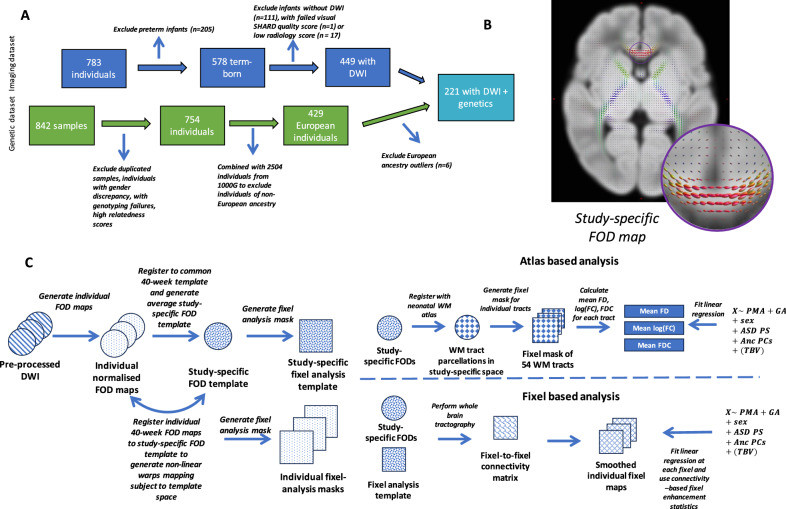
Participant selection and diffusion data preprocessing. **A** Flowchart of selection of participants. **B** Flowchart of diffusion data preprocessing and subsequent atlas-based and fixel-based analysis. **C** Study-specific FOD map. The circle shows the FOD orientation in the genu of the corpus callosum.

Participant selection is summarised in Fig. 1A. Of the total 783 individuals in the dHCP, only term-born (born at least 37 weeks of GA) infants with available diffusion-weighted imaging (DWI) data were selected (n = 467). This was to account for any possible effect of prematurity on WM development [32]. Multi-shell high angular diffusion imaging (HARDI) data were obtained using a protocol optimised for the neonatal brain as previously described [33, 34]. Briefly, infants' brain scans were obtained during their natural sleep using a dedicated neonatal brain imaging system on a 3T Philips Achieva scanner. The bespoke imaging system deposited infants in a standardised pose with adjustments only in the head-to-foot direction at the start of scanning. The field of view was set to accommodate 95% of late-term neonates [31, 35]. The scanning software was optimised to gradually ramp up gradient waveforms over 5 s before starting each sequence to prevent waking the infants. The full imaging protocol included the acquisition of calibration scans, anatomical images (T1w and T2w), resting-state functional and diffusion MRI at an average rate of 27 slices per second and a total scan duration of 1 h 3 min and 11 s. Diffusion MRI data were acquired using a pulsed-gradient spin-echo echo planar imaging (PGSE EPI) sequence with multiband factor 4, time to echo (TE)/ repetition time (TR) = 90/3800 ms, 1.5 × 1.5 × 1.5 mm resolution, sensitivity encoding (SENSE) 1.2, Partial Fourier 0.855 was used to yield 300 diffusion-weighted volumes per subject with four phase-encoding directions: b = 0 s/mm2 (n = 20), b = 400 sm/mm^2^ (n = 64), b = 1000 s/mm^2^ (n = 88) and b = 2600 s/mm^2^ (n = 128), where n is the number of diffusion-weighted directions.

DWI processing was done as part of the dHCP pipeline [31]. Briefly, images were first denoised [36] and corrected for Gibbs ringing artefacts [37]. Motion and image distortion were corrected using spherical harmonics and radial decomposition (SHARD) [38] (n = 18 individuals excluded after failed SHARD reconstruction quality check and low radiology score (the images were scored by an expert radiologist from 1–5 with 5 denoting images of the lowest quality). Subsequently, inter-slice intensity variations were adjusted [39], and individual brain masks were then generated using the *FSL* brain extraction tool (BET) [40]. Visual inspection of DWI and mask outputs was carried out by HL to remove any outliers with distorted brain masks (no outliers).

T2-weighted images were acquired using turbo spin echo (TSE) sequence parameters TR = 12 s, TE = 156 ms, and resolution (mm) 0.8 × 0.8 × 1.6. A series of motion correction [41] and super-resolution reconstruction techniques [42] were then employed to produce images of resolution (mm) 0.5 × 0.5 × 0.5. Subsequently, T2-weighted images were segmented with Developing brain Region Annotation With Expectation-Maximization (DrawEM) neonatal segmentation algorithm [43] as part of the dHCP pipeline [44]. Briefly, the algorithm utilises an intensity-based segmentation approach and a priori brain structural information from manually segmented atlases [45] to partition each subject’s brain image into 87 subcortical and cortical regions. The full detail of the algorithm is described in [43]. Total brain volume (TBV) was calculated as the sum of the volumes of cortical white and grey matter (GM), deep GM, cerebellum, and brain stem. The volumes were calculated as the total number of voxels multiplied by the voxel dimension.

### Genetic data preprocessing

Saliva samples were collected either at infants’ initial MRI sessions or at 18-month timepoints or both using the Oragene DNA OG-250 kit. Of the total 842 saliva samples, only one per individual was retained (usually the first one) for genotyping on the Illumina Infinitum Omni 5-4 v1.2 array. The genetic data quality control and preprocessing steps used in this study are described in Cullen et al. [46] (Fig. 1A). Briefly, excluded were genotyped data based on the following criteria: completeness of less than 95%, gender discrepancy and genotyping failure of more than 1% of the single nucleotide polymorphisms (SNPs). If the relatedness score between any individual pair was above a cut-off (pi_hat > = 0.1875), only one sample was randomly retained. Genotyped SNPs were filtered based on the following criteria: being non-autosomal, having minor allele frequency less than 0.05, missing in more than 1% of individuals or deviating from Hardy-Weinberg equilibrium with a p-value < 1 × 10^−5^.

Subsequently, the dataset was imputed to the Haplotype Reference Consortium reference panel [47] on the Michigan Imputation Server. The VCF files returned were converted to PLINK files using a genotype calling threshold of 0.9. The imputed SNPs were excluded based on the following criteria: having minor allele frequency less than 0.05, missing in more than 1% of individuals, deviating from Hardy-Weinberg equilibrium with a p-value < 1 × 10^−5^, or having an imputation R^2^ value of less than 0.8. All quality control was performed with PLINK 1.9. This yielded 754 individuals with high-quality SNPs.

### Population stratification

Ancestry subpopulations were identified by merging our cohort with 2504 individuals from the 1000 Genomes project [48] using a subset of common autosomal SNPs. Principal component analysis (PCA) was then performed on the resulting genetic dataset with PLINK, and the resulting principal components (PC) were then used to visually assign infants to specific ancestral subpopulations. Since the discovery sample used to derive the genome-wide association study (GWAS) summary statistics included exclusively individuals of Danish ancestry, only infants of European ancestry were considered for the ongoing analysis. Here, 429 (57%) were determined to have European ancestry.

European ancestry PCs were then generated from the infants of European ancestry subpopulation genetic data, and a visual examination of PC pairwise scatterplots was carried out to exclude ancestral group outliers (6 excluded). Of the remaining individuals, 221 individuals were term-born and had both imaging and genetic data available (Table 1).

**Table 1:** Demography of the 221 infants studied in this study.

Sex (male/female)	111/110
Gestational age at birth (week)	40.1 ± 1.2
Postmenstrual age at scan (week)	41.6 ± 1.7
Total Brain Volume (mm^3^)	376,314 ± 48,579

### Polygenic score calculation

Individual polygenic scores (PS) were calculated using PRSice-2 [49] software and summary statistics derived from the largest to-date autism GWAS [25, 50]. Here, PS for each infant were estimated at 10 p-value thresholds (P_T_): 10^−8^, 10^−6^, 10^−5^, 0.0001, 0.001, 0.01, 0.05, 0.1, 0.5, and 1, such that each score was composed of only those SNPs with autism GWAS association p-value less than the respective threshold. Genotype data of European individuals in the 1000 Genomes project was used as the external linkage disequilibrium reference panel [48].

### Fixel-based analysis preprocessing

The FBA framework [21], adapted to the neonatal cohort as described in Lautarescu et al. [51], was implemented in *MRtrix3 v.3.0.4* [52] to derive WM phenotypes examined in this study. Unlike adult HARDI, where there is a clean tissue separation based on distinct b-values, neonatal data does not provide a clear separation between WM and GM. For example, at term-equivalent age, the average signal from cortical GM is indistinguishable from that in corpus callosum [53]. Here, single-fibre WM and cerebrospinal fluid (CSF) tissue-specific response functions were calculated for each subject using the ‘dhollander’ method [54] with a tissue separation fractional anisotropy (FA) threshold of 0.15. This method leverages information from all diffusion-weighted shells acquired [55]. Response functions representative of CSF and WM diffusion signals at 44 weeks were calculated by averaging all CSF and 21 WM response functions from subjects aged 44.1 weeks, respectively. Subsequently, the average response functions were used to compute individual WM fibre orientation distributions (FODs) maps using the multi-shell multi-tissue constrained spherical harmonics method [56] and the estimated FODs were intensity normalised [57, 58]. The MSMT CSD method was performed on all diffusion-weighted shells.

To generate the study-specific FOD template, individually normalised FODs and masks were first registered from native to the 40-week anatomical template [59] using structural registration. The resulting warps were provided as part of the dHCP extended-release 3 [31]. Briefly, the warps were created from stepwise registrations as follows: subject DWI to subject T2-weighted image (*FSL FLIRT* using a normalised mutual information metric), subject T2-weighted image to age-matched T2-weighted template (using symmetric normalisation (SyN) algorithm from Advanced Normalisation Tools (ANTs) [60] and nonlinear diffeomorphic multimodal registration of T2-weighted image and GM/WM tissue probability maps) and week-to-week nonlinear transformations to the 40-week dHCP extended atlas (*ANTs SyN*). Consequently, the individual FODs and masks were transformed to the 40-week anatomical template (using the structural registered warps) using cubic and linear interpolation, respectively. The study-specific mask and FODs templates were then created by computing the intersection of all subject 40-week masks and the average of all subject 40-week WM FODs, respectively, and regrided to 1.3 mm isotropic voxel dimension. Finally, subject 40-week warped FODs images were registered to the study-specific FODs template (resulting in nonlinear warps mapping subject to template space) and then transformed to the template space without FOD reorientation (as previously explained in Raffelt et al. [21]).

### Fixel-wise metrics

A fixel analysis mask refers to a fixel grid on which fixel-related metrics are mapped and statistical analysis carried out. Here, a study-specific fixel analysis mask was generated by directly segmenting study-specific WM FODs using a peak amplitude threshold of 0.06 [61].

The amplitude of the WM FOD, which is proportional to the radial diffusion signal, provides a measure of the volume fraction of the fibres, or apparent fibre density (FD), along the corresponding fibre orientation [21]. Subject-wise fixel analysis masks were generated from individual WM FOD maps (warped to template space but not reoriented) and fixel-wise FD was computed as the integral of the corresponding FOD lobe [62]. Subsequently, subject-wise fixels were reoriented using the local angular transformation obtained from the subject-to-template warp. To achieve effective comparison between individuals, each subject-wise fixel and its corresponding FD value were assigned to a unique fixel on the study-specific template using a spatial correspondence algorithm with a maximum angle threshold of 45°.

While fixel-wise FD measures local changes in density within a fixel, it does not capture volume changes across a fibre bundle as a whole, whose width could stretch over multiple fixels [18]. Similar to tensor-based morphometry, fixel-based morphometry utilises subject-to-template warp to identify volume changes. However, it specifically measures local volume differences across the bundle, perpendicular to the fixel orientation, excluding changes due to the fibre bundle length [21]. Here, fixel-wise fibre bundle cross-section (FC) was computed using the subject-to-template warp and study-specific fixel analysis mask, and FC > 1 and FC < 1 denoted local relative expansion and contraction, respectively.

Finally, fibre density and cross-section (FDC) was calculated as the fixel-wise product of FC and FD. This metric reflects the combined volume change manifested as either within-voxel FD, macroscopic FC or a combination of both [21].

### Atlas registration and mean tract metrics extraction

**Fig. 2:**
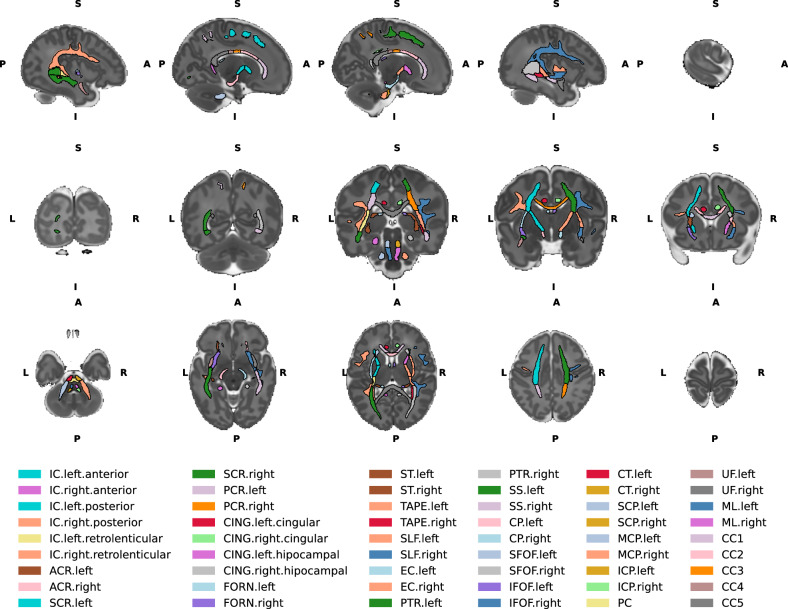
Neonatal white matter atlas. Top row - Left to right sagittal view of the brain slices. Middle row- Posterior to anterior coronal view of the brain slices. Bottom row - Inferior to superior axial view of the brain slices. IC internal capsule, ACR anterior corona radiata, SCR superior corona radiata, PCR posterior corona radiata, CING cingulum, FORN fornix, ST stria terminalis, SLF superior longitudinal fasciculus, EC external capsule, PTR posterior thalamic radiation, SS sagittal stratum, CP cerebral peduncle, SFOF superior fronto-occipital fasciculus, IFOF inferior fronto occipital fasciculus, CT corticospinal tract, SCP superior cerebellar peduncle, MCP middle cerebellar peduncle, ICP inferior cerebellar peduncle, PC pontine crossing, UF uncinate fasciculus, ML medial lemniscus, CC1 corpus callosum prefrontal part, CC2 premotor/supplementary motor part, CC3 corpus callosum motor part, CC4 corpus callosum sensory part, CC5 corpus callosum parietal/temporal/occipital part. L- left, R- right, A- anterior, P- posterior, S- superior, I- inferior.

Neonatal WM tract parcellations and the fibre orientation distribution function (ODF) template at 40 weeks were obtained from Uus et al. (https://gin.g-node.org/alenaullauus/4d_multi-channel_neonatal_brain_mri_atlas) [63] (Fig. 2). Firstly, the study-specific FOD template was registered to the neonatal atlas ODF template at 40 weeks using FOD registration to obtain non-linear deformation warps. Secondly, a binary mask for each of the 54 WM tract parcellations was generated from the neonatal WM tract parcellation. Thirdly, the individual binary mask was transformed to the study-group space using warps and linear interpolation, where voxels were assigned to the tract with the highest interpolated value. Fourthly, a fixel analysis mask for each WM tract was then created by assigning all fixels within the voxel of the tract. Finally, average fixel-wise FD, log(FC) and FDC values were generated for each WM tract label in each individual.

### Whole-brain tractography

Whole brain anatomically constrained probabilistic tractography was performed on the study-specific average FOD map to produce 20 million streamlines [64, 65]. To improve the biological plausibility of the tractography process, the tractogram was subsequently filtered using the SIFT algorithm to match streamline density to the diffusion signal, resulting in a reduced subset of 2 million streamlines [61]. The fixel template and the reduced subset of streamlines were then used to generate a fixel-to-fixel connectivity matrix. In turn, this allowed for the smoothing of fixel measures across connected fixels and improved the power of subsequent fixel-wise statistical analysis [57].

### Statistical analysis

In the first analysis, the association between autism PS and tract-mean FD, log(FC) and FDC was estimated using linear regression models. Here, dependent variables were the mean fixel-wise measures, and independent variables were sex, GA, postmenstrual age at scan (PMA), first 3 ancestry PCs, and autism PS. For examinations of the mean log(FC) and FDC, TBV was also included as a covariate (i.e., X ~ sex + GA + PMA + AncPCs + autism PS + (TBV), where X is mean tract FD, log(FC) or FDC). The variance R^2^ explained by the autism PS was reported as the difference between the R^2^ of the full model with the autism PS included as a covariate and the R^2^ of the base model without the autism PS included as a covariate. The statistical analysis was performed using the *statsmodels* module v 0.13.2 (https://www.statsmodels.org/stable/index.html) in Python.

Since PS across the 10 examined P_T_ were highly correlated, a method to utilise eigenvalue variance was used to calculate the effective number of independent tests performed [66]. The resulting number of independent tests for PS was 6. Given that FDC is a multiplication of FD and FC, the number of independent tests for all fixel-wise metrics was established as 2. Therefore, the multiple-comparison Bonferonni adjusted P-value threshold for examination of mean fixel metrics across 54 tracts was determined as 0.05/(54 × 6 × 2) = 7 × 10^−5^.

In the second analysis, the same linear regression model was fitted at each fixel in the fixel analysis mask (i.e., for whole brain WM: X ~ sex + GA + PMA + AncPCs + autism PS + (TBV), where X is fixel-wise FD, log(FC) or FDC). Here, connectivity-based fixel enhancement (CFE) was employed to assess the association between autism PS and fixel measures. CFE employs a threshold-free cluster enhancement algorithm to increase test statistics to structurally connected fixels [67, 68]. Contrast matrices were created to test for either negative or positive association between autism PS and fixel-wise measures. The design matrix was created by standardising the covariates with an added column of ones as intercept. The null distribution was generated by recording the maximum CFE statistics at each of the 5000 permutations and a fixel is considered nominally significant if the whole-brain two-tailed family-wise error (FWE) corrected p-value < 0.025 (0.05/2). Accounting for all PS P_T_ and fixel-wise measures yielded the whole-brain multiple-comparison Bonferonni adjusted P_FWE_ < 0.025/(6 × 2) = 0.002.

### Exploratory gene-set enrichment analysis

Exploratory gene-set enrichment analysis was performed on the autism SNP subset most associated with any WM mean tract fixel metric. Here, SNPs contributing to the autism PS threshold most strongly associated with the imaging phenotype were selected. Linear regression was performed for each SNP (i.e., WM mean tract fixel measure ~ TBV + GA + PMA + sex + ancestry PCs) to determine its association with the WM phenotype. Subsequently, the SNPs with p-value < 0.05 were retained for further analysis, as they were reasoned to be associated with both autism and the WM phenotype. To determine whether the SNPs converged on a common biological pathway, genes containing those SNPs were examined for their functions. Here, each SNP was mapped to a single gene if its base pair location was found within the start and stop coordinates of the gene according to the human genome build 37 (https://ctg.cncr.nl/software/magma). Consequently, the resulting gene list was functionally tested against a curated database of 13 159 gene sets (pathways) obtained from MSigDB v.7.5.1 (curated canonical pathways from Reactome, KEGG, Wikipathways and Gene Ontology; https://www.gsea-msigdb.org/gsea/msigdb/). A pathway was considered enriched (overrepresented) if the probability of observing the pathway-related genes in the user input gene list was different from random chance [69]. Hypergeometric test as implemented in GENE2FUNC on the FUMA platform [70] (https://fuma.ctglab.nl/gene2func) was carried out using 19 427 genes from the human genome Build 37 as background genes. Enriched pathways (Bonferroni corrected p-value < 0.05/13 159 = 3.79 10^−6^) with at least 4 overlapping genes with the gene list were reported. To ensure consistency and reproducibility of the results, two additional tests were carried out. In the first test, we randomly selected from the SNP subset that contributed to autism PS P_T_ = 0.01 the same number of SNPs that were associated with the WM phenotype of interest and performed the hypergeometric method. This test was simulated 1000 times, and the most enriched pathway in each run was recorded. Pathways specific to the WM phenotype were those that were observed in less than 5% of all random experiments. In the second test, we repeated our analysis with other similar bioinformatic tools, including DAVID v.2021 (http://david.abcc.ncifcrf.gov/) [69, 71] and WebGestalt v. 2019 (http://www.webgestalt.org/) [72] to confirm the FUMA findings.

## Results

### Atlas-based analysis

**Fig. 3:**
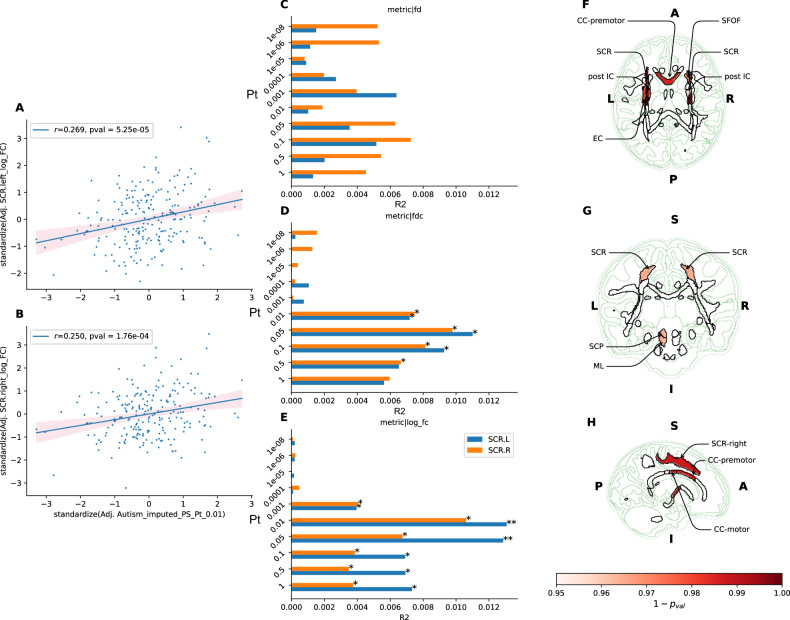
Atlas-based analysis. **A**–**B** Scatter plots of association between mean log FC of left and right superior corona radiata and autism-PRS at P_T_ 0.01. **C**–**E** Bar plots of R-squared of mean fixel metrics explained by autism PS across different P_T_. * p < 0.05 ** p < 7.7 × 10^−5^. **F**–**H** Visual representation of tracts with mean log FC associated with autism-PRS at P_T_ 0.01. (CC corpus callosum, SCR superior corona radiata, SFOF superior fronto-occipital fasciculus, post IC posterior internal capsule, EC external capsule, ML medial lemnicus, SCP superior cerebral peduncle.

Statistically significant associations between mean log(FC) and autism PS were identified in the regions of left superior corona radiata (SCR) (highest *R*^*2*^ = 0.013, standardised β = 0.11; *p-value* = 5.2 × 10^−5^ at PS P_T_ = 0.01) (Table 2; Fig. 3; Supplementary Table 1). Positive associations between mean log(FC) and several autism PS P_T_ were also identified in the regions of the right SCR (highest *R*^*2*^ = 0.010; standardised β = 0.10*, p-value* = 2.19 × 10^−4^ at PS P_T_ = 0.01) and the left posterior limb of the internal capsule (highest *R*^*2*^ = 0.008, standardised β = 0.09, *p-value* = 0.013 at PS P_T_ = 0.01), but the associations did not survive multiple testing correction.

**Table 2:** Standardised beta and p-value of associations between autism PS and WM tracts mean log(FC) across several PS P_T_.

P_T_	10^−8^	10^−6^	10-5	0.0001	0.001	0.01	0.05	0.1	0.5	1
Tract										
SCR.L	−0.014/6.28 × 10−^1^	−0.014/6.31 × 10−^1^	−0.013/6.70 × 10−^1^	0.010/7.38 × 10−^1^	**0.064/2.87** × **10**−^**2**^	^a^**0.117/5.55** × **10**−^**5**^	^a^**0.115/6.43** × **10**−^**5**^	**0.085/3.68** × **10**−^**3**^	**0.085/3.63** × **10**−^**3**^	**0.088/2.78** × **10**−^**3**^
SCR.R	−0.010/7.31 × 10−^1^	−0.015/5.85 × 10−^1^	0.003/9.28 × 10−^1^	0.022/4.39 × 10−^1^	**0.065/2.22** × **10**−^**2**^	**0.105/1.80** × **10**−^**4**^	**0.083/3.00** × **10**−^**3**^	**0.063/2.57** × **10**−^**2**^	**0.060/3.39** × **10**−^**2**^	**0.063/2.79** × **10**−^**2**^
CC-premotor	−**0.091/4.15** × **10**−^**2**^	−**0.101/2.04** × **10**−^**2**^	−0.074/9.59 × 10−^2^	0.014/7.49 × 10−^1^	0.083/6.09 × 10−^2^	**0.109/1.40** × **10**−^**2**^	0.066/1.34 × 10−^1^	0.042/3.41 × 10−^1^	0.070/1.17 × 10−^1^	0.073/1.04 × 10−^1^
PLIC.L	0.043/2.31 × 10−^1^	0.007/8.52 × 10−^1^	−0.008/8.20 × 10−^1^	0.011/7.64 × 10−^1^	0.043/2.37 × 10−^1^	**0.092/1.02** × **10**−^**2**^	**0.081/2.36** × **10**−^**2**^	**0.071/4.79** × **10**−^**2**^	**0.071/4.77** × **10**−^**2**^	**0.074/3.90** × **10**−^**2**^

Columns denotes varying thresholds of PS P_T_ and rows denote individual WM region. Selected are WM tracts with more than 2 associations with p-value < 0.05. **Bolded** are associations with p-value < 0.05.

*SCR.L* left superior corona radiata, *SCR.R* right superior corona radiata, *CC-premotor* corpus callosum / premotor and supplementary motor part, *PLIC.L* left posterior limb of the internal capsule.

^a^denotes statistically significant associations with p-value < 7 × 10^−5^.

No statistically significant association was found between FD or FDC and autism PS at any P_T_ in any of the tracts examined. Positive associations were identified between mean FDC and autism PS in the regions of left SCR (highest *R*^*2*^ = 0.01; standardised β = 0.01, *p-value* = 0.01 at PS P_T_ = 0.05), right SCR (highest *R*^*2*^ = 0.009, standardised β = 0.09, *p-value* = 0.01, PS P_T_ = 0.05), left external capsule (highest *R*^*2*^ = 0.014, standardised β = 0.12, *p-value* = 0.004, at PS P_T_ = 0.01), and left superior longitudinal fasciculus (highest *R*^*2*^ = 0.01, standardised β = 0.10, *p-value* = 0.01 at PS P_T_ = 0.5) (Supplementary Fig. 1–2). No nominal association (p-value < 0.05) was identified between mean FD and any of the tracts examined.

### Whole-brain fixel-wise analysis

**Fig. 4:**
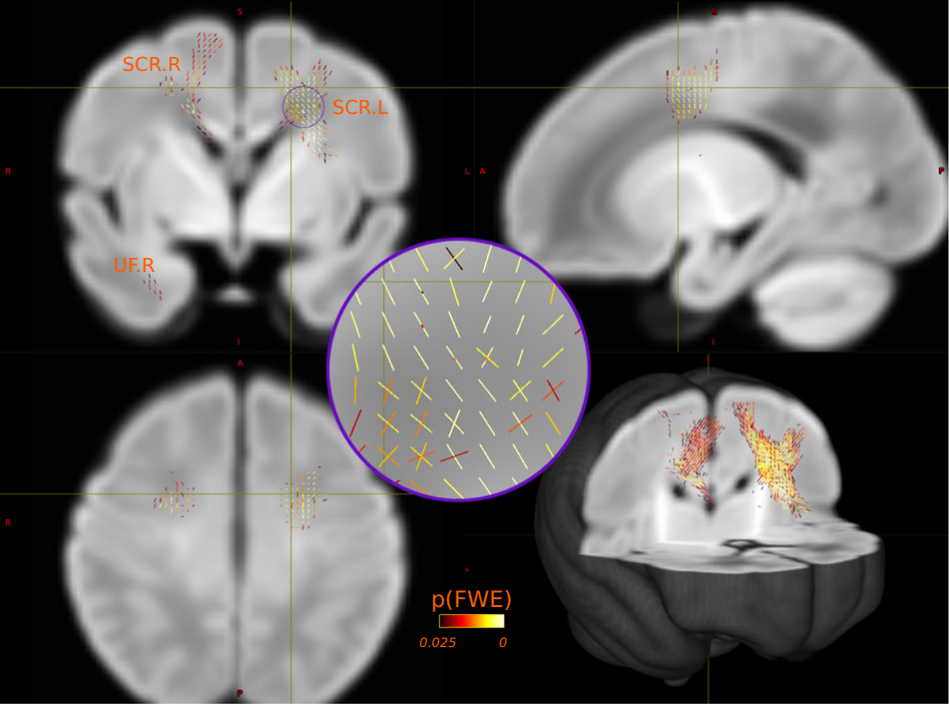
Fixel-wise association between log(FC) and autism PS P_T_ = 0.01. Visualised are fixels with p_FWE_ < 0.025 (i.e., not corrected for all PS P_T_ and fixel metrics examined); higher autism PS associated with higher log(FC) value. *Inset:* Visualisation of individual fixels. *Bottom right*: 3D rendering of the study template and the relative position of the significantly associated fixels. SCR.R right superior corona radiata. SCR.L left superior corona radiata. UF.R right uncinate fasciculus.

Positive associations between log(FC) and PS P_T_ 0.01 were identified in fixels within the regions of the left (lowest uncorrected fixel p-value = 0.0003) and right SCR, the body of the corpus callosum and the right uncinate fasciculus, where higher log(FC) was associated with autism risk (Fig. 4). However, these associations did not survive multiple testing corrections when accounting for all autism PS P_T_ and multiple fixel metrics. A similar direction of association was identified between fixel-wise log(FC) and PS P_T_ 0.05 in the region of the left SCR. Positive associations between fixel-wise FDC were also identified with PS P_T_ 0.05 in the middle segment of the corticospinal tract, where higher FDC values were associated with higher PS (Supplementary Fig. 3). However, these associations also did not survive multiple testing corrections. No significant association was identified between FD and any autism PS value. No significant negative association was identified between autism PS and any fixel measure.

Since FC is a macroscopic measure of change in WM volume, we tested if a similar pattern of variation is also detected at the voxel-wise level using equivalent tensor-based morphometry analysis [73] (Supplementary Information 1). Here, we found positive associations between log(Jacobians) and autism PS P_T_ = 0.01 in the similar regions of left and right SCR, where higher autism risk was associated with greater volume expansion (Supplementary Fig. 4).

### Exploratory gene-set enrichment analysis

**Fig. 5:**
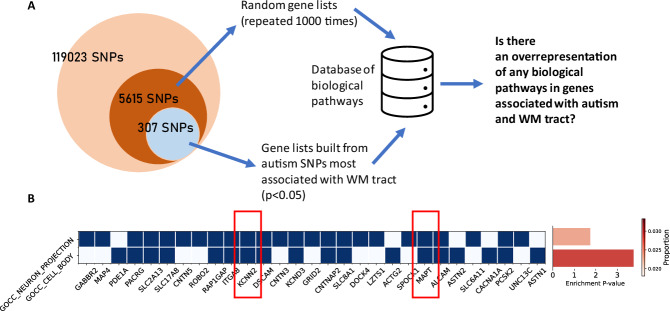
Gene-set enrichment result analysis. **A**. Visual flow chart of the exploratory gene-set enrichment analysis. **B**. Heatmap of genes involved in biological pathways enriched and specific (found in less than 5% across all random simulations) to the neuroimaging phenotypes of interest. Bar plot showing pathway enrichment p-value after Bonferroni correction and proportion of gene overlapped with the gene list. Highlighted in the red box are genes previously identified as significantly associated with autism in the discovery GWAS.

Since the most robust association we identified was between left SCR log(FC) and autism PS P_T_ = 0.01, we selected SNPs contributing to this PS threshold for further analyses. Of the 119 023 available SNPs (i.e., the intersection of SNPs in discovery GWAS and SNPs in our dataset following default clumping in PRSice-2), 5615 were found to contribute to the autism PS P_T_ = 0.01. Fitting linear regression identified 307 SNPs associated with left SCR log(FC) with p-value < 0.05, of which 132 were found in the start and stop coordinates of 126 unique genes. Subsequent exploratory gene-set enrichment analysis of the gene list and retaining only those found in less than 5% across all random simulations yielded two unique gene sets: neuron projection (Gene Ontology ID: 0043005; adjusted p-value = 0.018) and cell body (Gene Ontology ID: 0044297; adjusted p-value = 0.0001) (Fig. 5). Finally, gene sets enriched for similar functions were also identified using DAVID and WebGestalt tools (Supplementary Table 2 and Supplementary Table 3).

## Discussion

To our knowledge, this is the first study to examine the association between autism PS and WM structural variations in a general paediatric population at birth. Here, we identified a statistically significant association between autism PS and fibre bundle cross-section at the level of the left SCR. The direction of association was consistent with previous studies of autism in early life [2, 74], where greater brain volumes were associated with a higher likelihood of autism. The current result suggests WM structural alterations associated with autism common variants may be detected at birth.

### Comparison with previous examinations of autism-related WM structural variations in infants

Previous studies have generally reported larger WM volumes, most prominently in frontal and temporal regions, between 6 months and 5 years of age in infants and toddlers who were later diagnosed with autism [1–3, 5, 75, 76]. Concurrently, examination of individual WM tracts has also revealed increased volume in those connecting the frontal, temporal, and subcortical regions at 3 years of age in individuals later diagnosed with autism [7, 77]. Cumulatively, this suggests WM overconnectivity may be linked to the condition in early life [78]. In line with these observations, our work has identified FC changes associated with autism PS in frontal, temporal, and subcortical WM connections, where greater WM volume was associated with higher autism risk.

While the current work did not identify any microstructural variation associated with autism PS at birth, previous studies found elevated FA in multiple WM tracts in infants at elevated likelihood of autism in their first years of life [4, 6–9, 77]. FA reflects the degree of diffusion along the principal eigenvector and may serve as a microstructural surrogate for the fibre coherence and strength [78]. However, the measure has several inherent shortcomings including non-specificity to axonal properties and susceptibility to extra-axonal signals and fibre geometry [18]; which in turn may give rise to misleading conclusions. One study of only 7 autistic toddlers [6] reported an association between variation in q-space analysis measures, probability and displacement, and autism diagnosis. While this approach improves over the conventional DTI in quantifying diffusivity, it does not model separate individual fibre populations within a voxel [18]. Given the lack of methods to directly compare the reported diffusion metrics with fixel-wise measures [18] and the limited literature in this area, further examination of WM microstructure is required.

### Comparison with previous imaging-PS studies

The current work also contributes to the small literature examining associations between autism common genetic variants and brain morphology in general paediatric populations. Previously, studying the same infant cohort, we identified associations between autism PS and regional lobe volumes, where higher autism risk was associated with larger frontotemporal but lower parieto-occipital volumes [79]. Building on those results, our current results indicate possible associations between autism PS and WM tracts in the frontal area.

To our knowledge, links between autism PS and early WM features have been studied in only two other cohorts of young children and adolescents between 3–21 years of age [80] and 9–12 [81]. While autism PS was not found to be associated with total WM volume [81], higher autism PS was associated with lower structural connectivity (as measured by streamline counts) between the right precentral gyrus and left precuneus and postcentral gyrus [80]. Taken together, results to date suggest that autism common variants may have the greatest effect on regional rather than global WM volume.

### Clinical relevance of superior corona radiata

The SCR contain projection fibres that connect the cerebral cortex and the spinal cord. Structural changes and lesions in this region have been previously linked to motor and cognitive impairments in adolescents and adults [82, 83]. Increasing lines of evidence suggest differences in both motor and cognitive development associated with autism may be detectable as early as the first few years of life [84–86]. For instance, infants at higher risk of autism, compared with those at lower risk, may exhibit more pronounced deficits in social communication such as attention, eye gaze and facial expression directed to others between 7 and 12 months of age [87]. Similarly, by 6 months of age, the same infants may exhibit reduced fine (e.g., object manipulation) and gross (e.g., walking) motor control skills compared with those at lower risk [84, 85, 88]. Motor behaviour between 6–9 months appears to be strongly predictive of expressive and receptive language at two years of age, raising the possibility that very early motor development may be associated with future language skills [85, 88].

Recently, motor and language abilities have also been associated with autism common variants in general paediatric populations [89, 90]. Evidence suggests higher genetic risk of autism was associated with lower muscle tone at 9–20 weeks [89] and lower gross motor and receptive language scores at 18 months [90]. Since dHCP also provides neurodevelopmental outcomes at 18 months (as measured by the Bayley Scale of Infant Development Third Edition [91]), we tested whether a similar relationship between autism PS and neurodevelopmental scores was also found in our sample of term-born infants of European ancestry (Supplementary Information 2). Here, we revealed autism PS was statistically associated with gross (standardised β = −0.44, p-value = 0.0005 at P_T_ = 0.0001) and combined gross and fine motor scores (standardised β = −0.70, p-value = 0.0005 at P_T_ = 0.0001), where higher autism PS was associated with lower motor scores (Supplementary Fig. 5). Similar negative trends between several autism PS and cognitive (standardised β = −0.32, p-value = 0.009 at P_T_ = 0.0001) and expressive communication scores (standardised β = −0.38, p-value = 0.012 at P_T_ = 0.0001) were also identified, but the associations did not survive multiple testing corrections. Finally, subsequent mediation analysis did not find the effect between autism PS and motor scores to be modulated by the mean log(FC) of the left SCR. Nevertheless, in agreement with previous literature, our result suggests there may be shared genetic susceptibility between autism and early motor development [89].

FC denotes the change in the cross-sectional area ascribed to the bundle of interest, in the direction *perpendicular* to the fibre orientation. This is distinct from the FD, which relates to the voxel-wise intra-axonal volume occupied by the axons along the direction of the bundle. The combination of FD (the voxel-wise estimate of fibre density) and FC (the change in the overall cross-sectional area of the bundle) therefore provides a measure of the overall cross-sectional area taken up by the axons across the bundle as a whole. This metric, labelled FDC, can be thought of as a relative measure of *the* *information-carrying capacity* of the bundle (also referred to as the *fibre bundle capacity* in Smith et al. [92]). For FC specifically, if we assume no change in the average axonal diameter or density, increased FC implies an increase in the number of axons in the bundle. FD and FC variations do not necessarily imply myelination changes, but they may accompany or follow such biologically realistic scenarios. Myelin itself is not readily detectible using diffusion MRI signal due to its short T2, implying its contribution to the signal is negligible due to the long echo times typically used. Nonetheless, myelin changes can affect the signal from the *extra-axonal* space, if only by the absence of signal from the volume it occupies, and this can in turn affect the parameters of diffusion MRI models. However, such parameters are also affected by other factors and cannot be specifically interpreted as myelin using diffusion MRI alone. It is also possible that increased myelin reduces membrane permeability, which would reduce the apparent radial diffusivity and thereby also increase the estimated apparent fibre density. However, this is likely to be a small effect, given that intact axonal membranes are sufficient to provide very high levels of anisotropy even in the absence of myelin [93].

### Exploratory gene-set enrichment analysis

Functional analysis of autism SNPs associated with SCR log(FC) revealed enrichment of both cell body and neuron projection. This is not surprising since many of the autism common variants appear to be found in genes with elevated expression during foetal corticogenesis and related to neuronal functions [25]. Nevertheless, the overrepresentation of neuron projection may support the possible involvement of disrupted connectivity in the pathophysiology of the condition. For example, many of the highlighted genes in that pathway have also been studied in animal and in vitro models of the condition for their role in synaptic formation and activity (e.g., *DSCAM* [94], *ASTN2* [95]). Interestingly, using this approach, we also identified two genes, *MAPT* and *KCNN2*, which were significantly associated with the condition in the latest GWAS [25]. In that study, *KCNN2*, encoding for the voltage-independent Ca^2+^-activated K^+^ channel, was the most strongly associated gene with autism that did not contain any genome-wide significant SNP [25].

### Limitation

The limitation of our study lies in the small sample size and simplicity of PS. Here, only term-born infants of European ancestry were included due to the known effect on brain imaging phenotype by prematurity and the inclusion of predominantly individuals with parents of Danish ancestry in the discovery GWAS [25]. Finally, although overrepresentation analyses may be biased towards larger well-studied pathways, the result provided may still indicate relevant avenues for further research.

## Conclusion

In conclusion, by examining fixel-wise measures in term-born infants, we identified a positive association between autism PS and left SCR FC; where greater autism PS was associated with a larger WM tract cross-sectional area. Whilst preliminary, the current work contributes to the limited literature exploring the role of autism common variants in the general paediatric population and the early emergence of this condition.

## Supplementary information

Supplementary Information

Supplementary Table 1

## Data Availability

The MRI data used (dHCP third release) is freely available: https://biomedia.github.io/dHCP-release-notes/. The described statistical analysis can be recreated using scripts provided here: https://github.com/lehai-ml/dHCP_genetics. Scripts used for visualising results in this study are available here: https://github.com/lehai-ml/nimagen.
