# Supplementary material for: Autism common variants associated with white matter alterations at birth: cross-sectional fixel-based analyses of 221 European term-born neonates from the developing human connectome project
Source: Transl Psychiatry. 2025 Feb 4;15:40. doi: 10.1038/s41398-025-03252-3 (PMC11794609; doi:10.1038/s41398-025-03252-3)
Supplement: Supplementary file 1 — Supplementary Information [file 41398_2025_3252_MOESM1_ESM.docx]

**Supplementary Information**

**Autism common variants associated with white matter alterations at birth: Cross-sectional fixel-based analyses of 221 European term-born neonates from the developing Human Connectome Project**

Hai Le MRes^1+^, Alexandra F. Bonthrone PhD^1^, Alena Uus PhD^1^, Daphna Fenchel PhD^1^, Alexandra Lautarescu PhD^1,2^, Konstantina Dimitrakopoulou PhD^3^, A. David Edwards DSc^1^, Joseph V Hajnal PhD^1^, Serena J. Counsell PhD^1^, Lucilio Cordero-Grande PhD^1,4^, Daan Christiaens PhD^1^, Dafnis Batalle PhD^1,2^, Maximilian Pietsch PhD^1^, Anthony N Price PhD^1^, Hamel Patel PhD^5^, Charles Curtis MRes^5,6^, Harriet Cullen PhD^1,7*^, Maria Deprez PhD^1*^& Jacques-Donald Tournier PhD^1*^

* Contributed equally

^1^ *Research Department of Early Life Imaging, School of Biomedical Engineering and Imaging Sciences, King’s College London, London, United Kingdom*

^2^ *Department of Forensic and Neurodevelopmental Sciences, Institute of Psychiatry, Psychology and Neuroscience, King’s College London, London, United Kingdom*

^3^ *Translational Bioinformatics Platform, NIHR Biomedical Research Centre*, *Guy’s and St. Thomas’ NHS Foundation Trust and King’s College London, London, United Kingdom*

^4^ *Biomedical Image Technologies, ETSI Telecomunicación, Universidad Politécnica de Madrid & CIBER-BBN, ISCIII, Madrid, Spain*

^5^ *NIHR BioResource Centre Maudsley, NIHR Maudsley Biomedical Research Centre at South London and Maudsley NHS Foundation Trust & Institute of Psychiatry, Psychology and Neuroscience, King’s College London, London, United Kingdom*

^6^ *Social Genetic & Developmental Psychiatry Centre, Institute of Psychiatry, Psychology and Neuroscience, King’s College London, London, United Kingdom*

^7^ *Department of Medical and Molecular Genetics, School of Basic and Medical Biosciences, King’s College London, London, United Kingdom*

**^+^ Correspondence:** [**L.haibg@gmail.com**](mailto:L.haibg@gmail.com) **/ 1^st^ Floor, South Wing, St. Thomas’ Hospital, London SE1 7EH, UK**


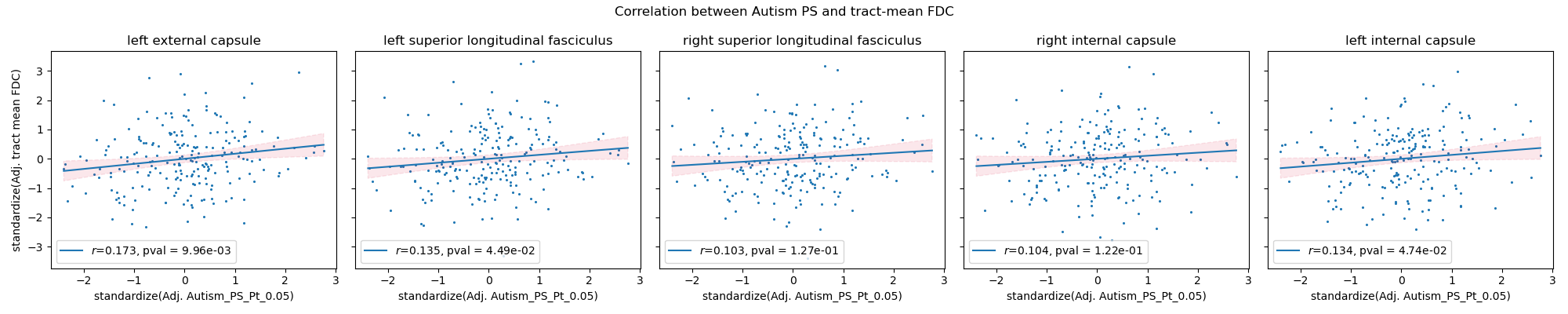


**Supplementary Figure 1.** Plot of correlation of the top 5 tracts with mean FDC value most associated with Autism PS P_T_=0.05. Here, the FDC value is adjusted for GA, PMA, TBV and sex and autism PS is adjusted for first 3 ancestry PCs


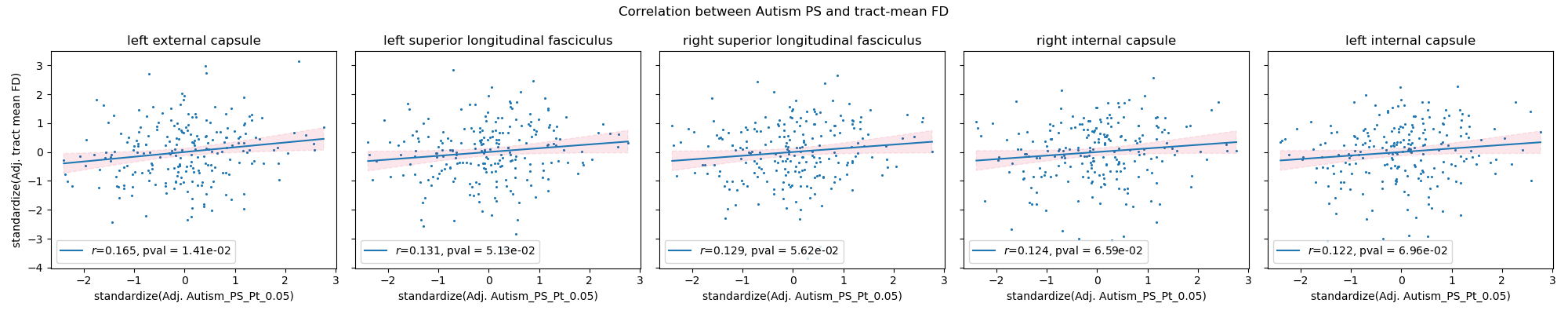


**Supplementary Figure 2.** Plot of correlation of the top 5 tracts with mean FD value most associated with Autism PS P_T_=0.05. Here, the FDC value is adjusted for GA, PMA and sex and autism PS is adjusted for first 3 ancestry PCs

**
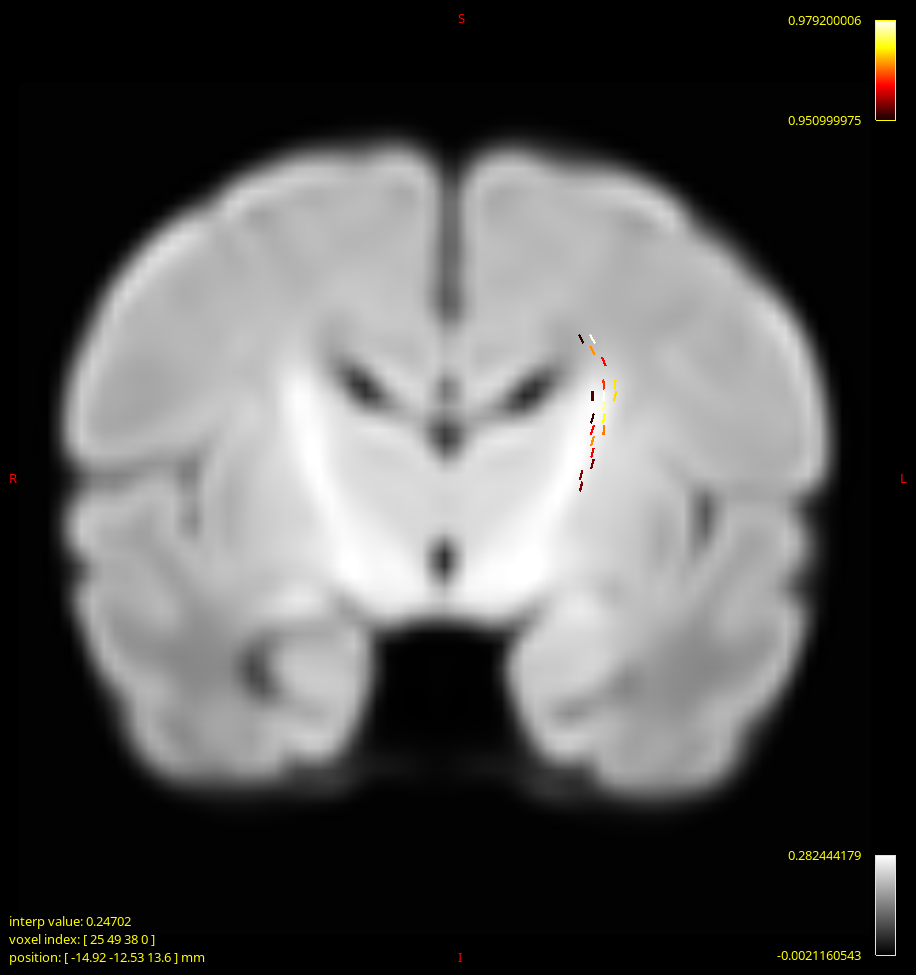
**

**Supplementary Figure 3.** Visualisation of the most associated fixel between FDC and Autism PS P_T_=0.05 mapped on an average group template. Colour scale of (1-p-value) is set from 0.95 to 1. Coronal section.

**Supplementary Information 1: Confirmatory analysis with log-Jacobians maps**

Log-Jacobians maps were derived as described here [Gale-Grant et al., 2022]. Briefly, motion correction and slice-to-volume reconstruction of the scans were performed as part of the dHCP pre-processing pipeline [Cordero-Grande et al., 2018]. Individual T2 weighted images were then registered to a dHCP week specific template (according to individual postmenstrual age at scan) on a 40-week common space [Schuh et al., 2018] using symmetric normalisation algorithm from Advanced Normalisation Tools version 3.0 [Avants and Gee, 2004]. Deformation tensor fields yielded were then used to compute scalar and, subsequently, logarithmic values of Jacobian determinants. The log Jacobians determinants maps were then smoothed with a sigma of 3.5 mm full width at half maximum Gaussian filter. Finally, voxel-wise examination of association between log(Jacobians) maps and autism PS using similar contrast and design matrices as in FBA were carried out using MRtrix3 *mrclusterstat* command.

Here, we found positive associations (one-sided P_FWE_ < 0.05) between log(Jacobians) and Autism PS P_T_ = 0.01 in the regions of left and right superior corona radiata (Supplementary Figure 1).


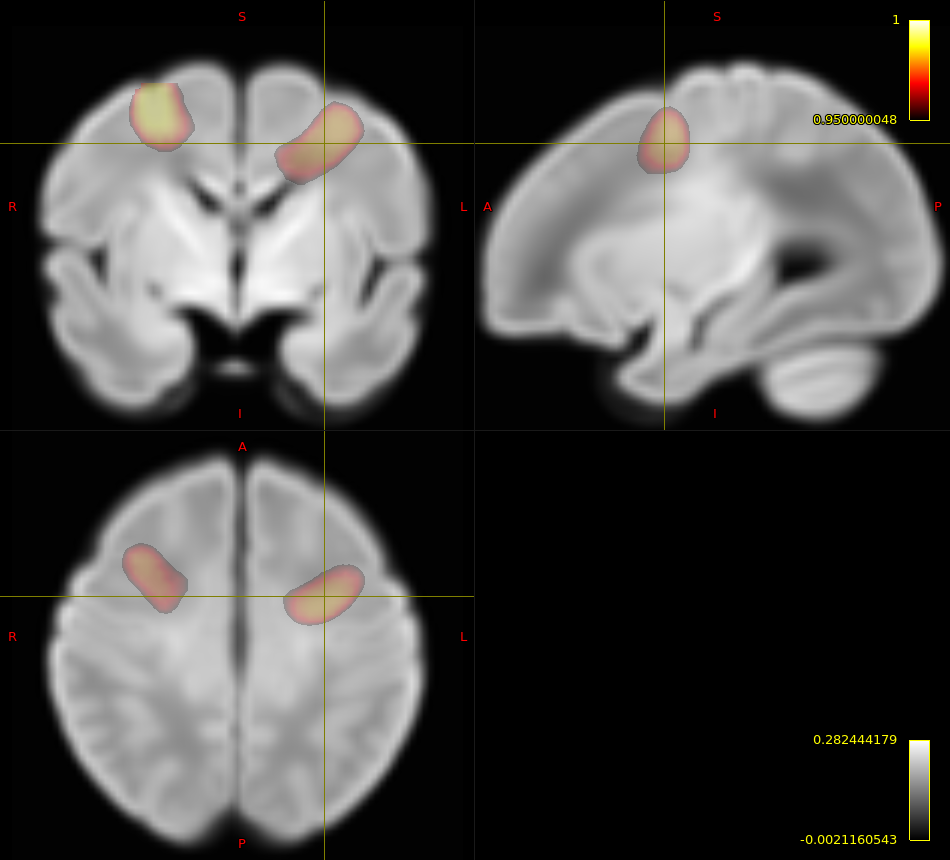


Supplementary Figure 4. Cluster of voxels showing statistically significant association between autism PS P_T_ = 0.01 and log(Jacobians determinant) in the positive direction (mapped on the 40-week common template). Hot colour bar denotes the association strength (1- p_FWE_) between 0.95 and 1.

**Supplementary Information 2: Behavioural outcome at 18 months**

Of the 293 European term-born infants with genotyped data, 249 individuals had follow-up behavioural outcomes assessment at 18 months. The outcomes measured were: cognitive, receptive communication, expressive communication, fine motor and gross motor. These measurements were derived using the Bayley Scale of Infant Development Third Edition [Albers and Grieve, 2007; Edwards et al., 2022] and were age-adjusted. The combined motor score was calculated as the sum of the fine and gross motor composite scores. The combined Communication score was calculated as the sum of the receptive and expressive communication composite scores. Index of multiple deprivation (IMD) score was generated for each infant using the parental postcode at the time of infant birth [Abel et al., 2016]. Linear regression model was fitted for each variable (fine, gross and combined motor scores, receptive, expressive and combined communication scores, and cognitive score) to assess its association with autism polygenic score (PS) at each PS threshold (PT), such that Behaviour score ~ sex + age at assessment + IMD + PS + first 3 Ancestry PCs. Method proposed by [Li and Ji, 2005] was employed to calculate the effective number of independent tests (Meff). For autism PS, this was calculated to be Meff = 6. Therefore, the multiple-comparison Bonferroni adjusted P-value threshold was determined to be p< 0.05/(6x7) = 0.0012.

Gross (standardised b = -0.44, p-value = 0.0005) and combined motor scores (standardised b = -0.70, p-value = 0.0005) were significantly associated with autism PS PT = 0.0001 (Supplementary Figure 5); where higher autism risk was associated with lower motor scores. Negative association (p<0.05) was also found between autism PS P_T_=0.0001 and cognitive score (standardised b = -0.32, p-value = 0.009) and expressive communications called score (standardised b = -0.38, p-value = 0.01). Negative associations (p<0.05) between gross and combined motor scores and autism PS was also found in autism PS= 1e-05,0.01 and 0.05. A trend towards a negative relationship was also detected between fine motor score and autism PS, but no significant association was found.


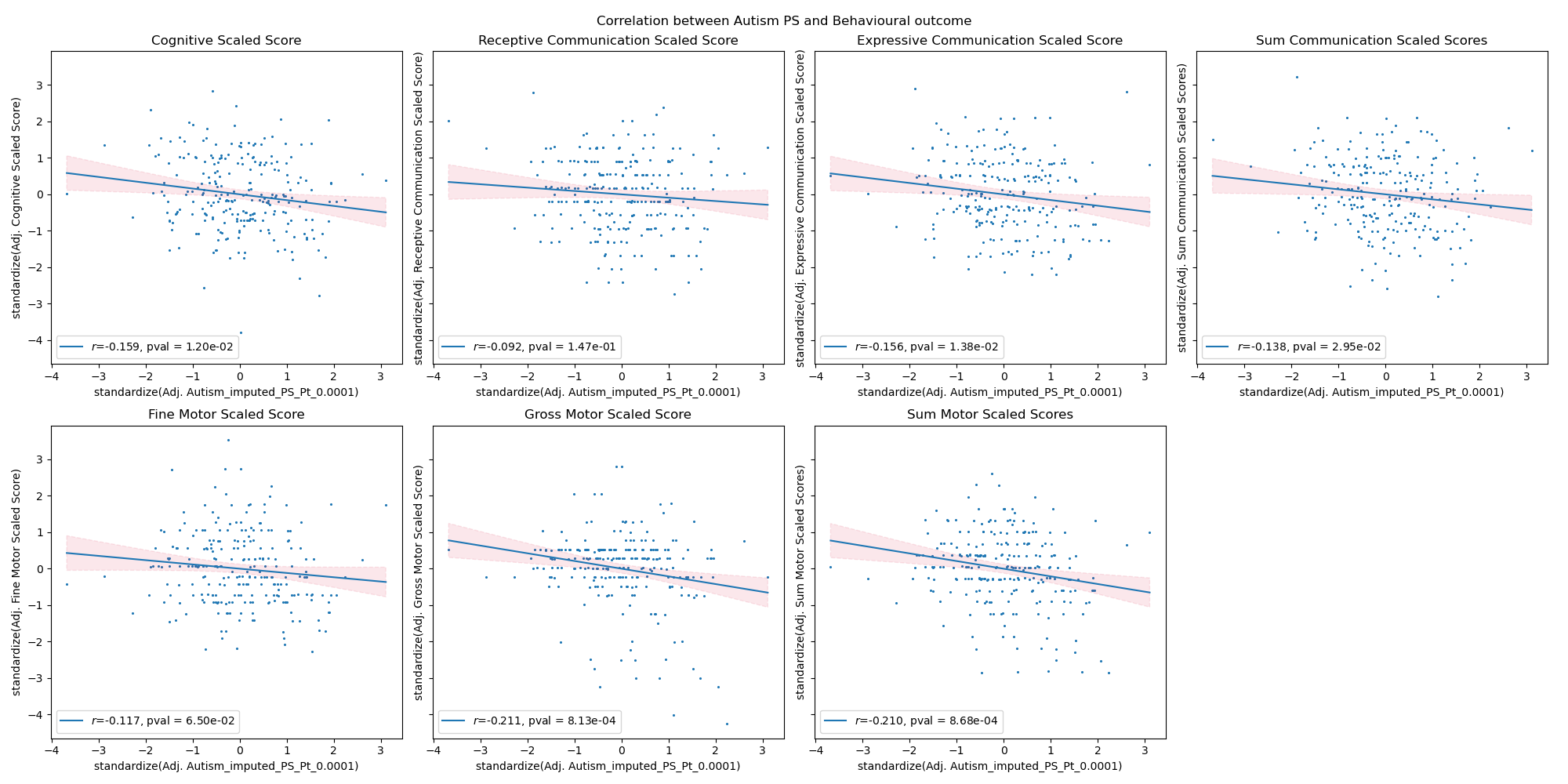


**Supplementary Figure 5**. Scatterplot between behavioural scores and autism PS P_T_ = 0.0001. Here, the assessment scores have been adjusted for sex, assessment age and IMD, and autism PS has been adjusted for the first 3 ancestry PCs.

**Supplementary Table 1.** *(See the accompanied xlsx file).* R^2^, β-value and p-value for each of the covariates in regression analyses involving left and right superior corona radiata. Highlighted are associations with p-value < 0.05.

**Supplementary Table 2**. Top 5 pathway identified by overrepresentation analysis with DAVID. **Bolded** are statistically significant results. The results were generated using “Homo Sapiens” as background genes; GO_CC_DIRECT, GO_MF_DIRECT and GO_BP_DIRECT as reference gene lists. Gene lists were uploaded as entrez IDs.

| Category | Term | Count | Proportion | P-value | Benjamini FDR |
| --- | --- | --- | --- | --- | --- |
| GO_CC_DIRECT | **Plasma membrane** | **52** | **41.3%** | **9x10^-5^** | **2.1x10^-2^** |
| GO_CC_DIRECT | **Neuronal cell body** | **10** | **7.9%** | **4x10^-4^** | **4.4x10^-2^** |
| GO_CC_DIRECT | Cell projection | 7 | 5.6% | 1.1x10^-2^ | 6.8x10^-2^ |
| GO_CC_DIRECT | Axon | 9 | 7.1% | 1.5x10^-3^ | 6.8x10^-2^ |
| GO_CC_DIRECT | Neuron projection | 9 | 7.1% | 2.6x10^-3^ | 8.7x10^-2^ |

**Supplementary Table 3**. Statistically significant results identified by overrepresentation analysis with WebGestalt. Human background genes of build 37 were uploaded as background genes. “geneOntology”: biological processes, molecular function and cellular components were used as reference gene lists. Gene lists were uploaded as entrez IDs.

| Gene Set | Description | Size | Exepct | Ratio | P-value | FDR |
| --- | --- | --- | --- | --- | --- | --- |
| GO:0097458 | neuron part | 1678 | 10.671 | 2.7177 | 4.5325e-7 | 0.0054916 |
| GO:0044297 | cell body | 549 | 3.4912 | 4.2965 | 0.0000020736 | 0.012562 |
| GO:0044456 | synapse part | 917 | 5.8314 | 3.2582 | 0.0000049639 | 0.020047 |
| GO:0045202 | synapse | 1148 | 7.3004 | 2.8766 | 0.000010182 | 0.030372 |
| GO:0043025 | neuronal cell body | 484 | 3.0779 | 4.2237 | 0.000012534 | 0.030372 |
